# Comparison of Glucosinolate Profiles in Different Tissues of Nine *Brassica* Crops

**DOI:** 10.3390/molecules200915827

**Published:** 2015-08-31

**Authors:** Shiva Ram Bhandari, Jung Su Jo, Jun Gu Lee

**Affiliations:** 1 Department of Horticulture, College of Agriculture & Life Sciences, Chonbuk National University, Jeonju 561-756, Korea; E-Mails: shivarbhandari@gmail.com (S.R.B.); jjs446@naver.com (J.S.J.); 2 Institute of Agricultural Science & Technology, Chonbuk National University, Jeonju 561-756, Korea

**Keywords:** *Brassica* crops, glucosinolates, root, seed, shoot, sprout

## Abstract

Glucosinolate (GSL) profiles and concentrations in various tissues (seeds, sprouts, mature root, and shoot) were determined and compared across nine *Brassica* species, including cauliflower, cabbage, broccoli, radish, baemuchae, pakchoi, Chinese cabbage, leaf mustard, and kale. The compositions and concentrations of individual GSLs varied among crops, tissues, and growth stages. Seeds had highest total GSL concentrations in most of crops, whereas shoots had the lowest GSL concentrations. Aliphatic GSL concentrations were the highest in seeds, followed by that in sprouts, shoots, and roots. Indole GSL concentration was the highest in the root or shoot tissues in most of the crops. In contrast, aromatic GSL concentrations were highest in roots. Of the nine crops examined, broccoli exhibited the highest total GSL concentration in seeds (110.76 µmol·g^−1^) and sprouts (162.19 µmol·g^−1^), whereas leaf mustard exhibited the highest total GSL concentration in shoots (61.76 µmol·g^−1^) and roots (73.61 µmol·g^−1^). The lowest GSL concentrations were observed in radish across all tissues examined.

## 1. Introduction

Many epidemiological studies have suggested that diets rich in fruits and vegetables are positively associated with human health in many ways, including reduced risk of cancers, type II diabetes, and cardiovascular diseases [1,2,3]. *Brassicaceae* vegetables, as a group, possess a large number of health-promoting compounds such as vitamins, carotenoids, flavonoids, polyphenols, minerals, and glucosinolates (GSLs) in considerable amounts [4,5,6,7,8,9,10]. Among these health-promoting phytochemicals, GSLs (β-thioglucoside-*N*-hydroxysulfates) are an important chemical group naturally occurring in almost all *Brassica* species. GSLs are a group of sulfur-containing glucosides that are hydrolyzed by the endogenous enzyme myrosinase into isothiocynates (ITCs), thiocynates, and nitriles [11]. More than 200 GSLs have been identified in *Brassica* crops, and they are characterized mainly by the variable R group, which can be aromatic, indolic, or aliphatic derivatives of the amino acid precursors methionine, tryptophan, and phenylalanine, respectively [12,13,14,15]. 

GSLs and their products have different biological functions, including anticancer, anti-bacterial, anti-fungal, anti-oxidative, and allelopathic properties [16,17,18]. For example, ITCs such as sulforaphane, iberin, phenylethyl, and prop-2-enyl, derived from glucoraphanin, glucoiberin, gluconasturtiin, and sinigrin, respectively, have been found to induce phase 2 enzymes promoting anti-proliferative activity [14,19]. GSLs can also be used as an alternative to synthetic pesticides for pest and disease control [20]. Some GSLs such as sinigrin and progoitrin are also responsible for the bitter flavor of these crucifers and influence consumer acceptance [14,21]; therefore, the determination of GSL concentrations in *Brassica* vegetables is important in the study of such activities in *Brassica* vegetables.

The content and profile of GSLs in *Brassica* vegetables significantly varies depending on various plant-specific factors such as the cultivar genotype, growth stage, or structure where GSL accumulates, as well as environmental conditions of temperature, light, water and nutrient availability, growing season, agricultural practices, and post-harvest conditions [4,5,7,22,23,24,25,26]. For example, variance in GSL concentrations was observed in the same broccoli genotype grown in different seasons and under different agricultural practices [25,27]. Thus far, most research work has focused on seed and fully mature plants [5,9,25,26]. Studies of GSL concentrations in sprouts are limited to a few members of *Brassicaceae*. Moreover, a paucity of research addresses GSL patterns and concentrations in the young shoots and roots of different *Brassica* species, in addition to the seeds and sprouts. *Brassica* crops such as cauliflower, broccoli, cabbage, radish, baemuchae, kale, pakchoi, Chinese cabbage, and leaf mustard are commonly used for various side dishes in many countries. Thus, investigation of GSL profiles and concentrations in crop commodities will be useful for consumers and agribusiness, in general. In addition, GSL profiling in roots, combined with seeds and other plant tissues, will improve our understanding of the distribution pattern of GSLs in whole plant tissues. Thus, in this study, we investigated GSL profiles of seeds, sprouts, roots, and shoots across nine widely consumed *Brassica* vegetables for the comparative analysis of GSL profiles.

## 2. Results and Discussion

### 2.1. Variation in GSL Concentration in Seeds

**Figure 1:**
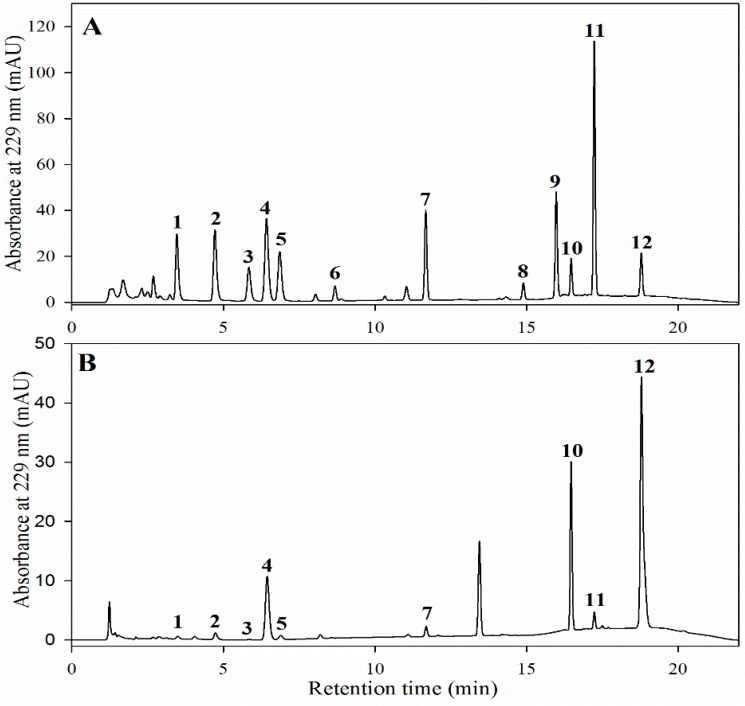
Ultra performance liquid chromatography (UPLC) chromatograms of desulfo-GSLs standards (**A**) and kale sample extract (**B**). Peak identification 1: glucoiberin; 2: progoitrin; 3: epiprogoitrin; 4: glucoraphanin; 5: sinigrin; 6: glucoraphenin; 7: gluconapin; 8: glucobarbarin; 9: glucobrassicanapin; 10: glucoerucin; 11: glucobrassicin; and 12: gluconasturtiin.

This study focused on examining the GSL profiles and their concentrations in various tissues and growth stages of nine *Brassica* crops. A total of 12 GSLs belonging to the three chemical classes were analyzed; these included nine aliphatic, one indolyl, and two aromatic GSLs. Identification and quantification of GSL peaks by chromatography were based upon the known concentrations of commercial standards and their retention time. A typical GSL chromatogram of standard mixture (A) and sample extract (B) is shown in Figure 1. Detailed information of individual desulfo-GSLs and their concentrations in the seeds of all nine *Brassica* crops examined were recorded (Table 1 and Table 2). These data show that the concentrations varied depending on the plant type and structure. The most commonly observed GSL was BRA, although it was present in lower quantities than the other GSLs.

**Table 1:** UPLC spectroscopy information on 12 identified major desulfo-glucosinolates studied in nine *Brassica* crops.

Serial Number	Trivial Name	Abbreviation	Chemical Group	Retention Time (min)	Semisystematic Name of Side Chain
1	Glucoiberin	IBE	Aliphatic	3.46	3-Methylsulfinylpropyl
2	Progoitrin	PRO	Aliphatic	4.72	2-Hydroxy-3-butenyl
3	Epiprogoitrin	EPI	Aliphatic	5.84	(2*R*)-2-Hydroxy-3-butenyl
4	Glucoraphanin	GRA	Aliphatic	6.42	4-Methylsulfinylbutyl
5	Sinigrin	SIN	Aliphatic	6.85	2-Propenyl
6	Glucoraphenin	GRE	Aliphatic	8.68	4-(Methylthio)butenyl
7	Gluconapin	NAP	Aliphatic	11.67	3-Butenyl
8	Glucobarbarin	BAR	Aromatic	14.89	2-Hydroxy-2-phenylethyl
9	Glucobrassicanapin	BCN	Aliphatic	15.98	4-Pentenyl
10	Glucoerucin	ERU	Aliphatic	16.47	4-(Methylthio)butyl
11	Glucobrassicin	BRA	Indolyl	17.23	3-Indolylmethyl
12	Gluconasturtiin	NAS	Aromatic	18.78	2-Phenylethyl

The dominant GSL varied according to the crop type and plant tissues examined. SIN was most abundant GSL in cauliflower (35.43 µmol·g^−1^), kale (66.49 µmol·g^−1^), and leaf mustard (80.44 µmol·g^−1^), whereas NAP was present in high concentrations in pakchoi (103.51 µmol·g^−1^), baemuchae (18.10 µmol·g^−1^), and Chinese cabbage (17.13 µmol·g^−1^). Similarly, broccoli, cabbage, and radish exhibited higher ERU (76.35 µmol·g^−1^), PRO (53.53 µmol·g^−1^), and GRA contents (0.90 µmol·g^−1^) than the other GSLs. GRE was only found in radish and baemuchae. These crop-dependent dominant GSLs were in good agreement with the results of previous studies [4,28]. Total GSL concentrations varied significantly among the crop types, with broccoli, cabbage, and pakchoi showing relatively higher total GSLs (<100 µmol·g^−1^) than the other crops. Radish seeds showed exceptionally lower total GSL content (2.45 µmol·g^−1^) across all crop types examined.

### 2.2. Variation in GSL Concentration in Sprouts

The GSL profiles and concentrations in nine-day-old *Brassica* sprouts were also compared. NAP, BRA, and NAS were the most common GSL present in all crops examined (Table 3). SIN was a major GSL in cauliflower (33.90 µmol·g^−1^), kale (55.56 µmol·g^−1^), and pakchoi (20.22 µmol·g^−1^), whereas ERU was a major GSL in broccoli (123.67 µmol·g^−1^), cabbage (34.88 µmol·g^−1^), and radish (1.25 µmol·g^−1^) sprouts, as found in seeds. ERU has both direct and indirect antioxidant effects [29]; therefore, the presence of ERU at a higher ratio in broccoli, cabbage, and radish suggested that these crops had more health benefits than the other crops examined.

Similar to seeds, GRE was only present in radish and baemuchae; however, the values in nine-day-old sprouts were relatively lower than those in seeds. Total GSL concentration also significantly varied from 2.46 µmol·g^−1^ in radish to 162.19 µmol·g^−1^ in broccoli. Only six of the 12 GSLs identified in this study were observed in radish; these include GRA, GRE, NAP, ERU, BRA, and NAS. Moreover, these GSLs, except GRE, were detected in very minute quantities as compared to those in other crops. In contrast, the highest number of GSLs (10 GSLs) was found in cabbage. Total GSL concentration was relatively lower in 9 day-old sprouts than in seeds of cabbage, Chinese cabbage, baemuchae, kale, pakchoi, and leaf mustard, whereas it was exceptionally higher in broccoli (162.19 µmol·g^−1^). Furthermore, cauliflower and radish exhibited very similar GSL contents in both seeds and nine-day-old sprouts.

### 2.3. Variation in GSL Concentration in Shoots

The GSL profile and concentrations in the shoots of *Brassica* crops also showed different accumulation patterns (Table 4) and the genotypic variation was statistically significant; this may be attributed to the difference in the regulatory mechanism of genes involved in GSL biosynthesis [15]. PRO, BRA, and NAS were the most common GSLs found in all the crops examined. Among these, BRA was the most dominant GSL in cauliflower (2.56 µmol·g^−1^), broccoli (1.44 µmol·g^−1^), cabbage (3.13 µmol·g^−1^), and Chinese cabbage (1.10 µmol·g^−1^), whereas NAP and SIN were the dominant GSLs in pakchoi (8.24 µmol·g^−1^) and leaf mustard (52.00 µmol·g^−1^), respectively. The highest concentrations of SIN observed in seeds and shoots was in good agreement with the results reported by Gupta *et al.* [30], who analyzed 97 lines of leaf mustard. The concentrations of PRO, which was found in relatively higher quantities in the seeds of most crops examined, were considerably reduced in shoots, except for in radish and leaf mustard. Similarly, ERU, a major GSL in broccoli and cabbage seeds was not present in broccoli shoot and its concentration was considerably reduced in cabbage shoots, which might be due to the catabolic processes in play during the development of plant tissue. In addition to the seeds and sprouts, shoots of radish and baemuchae possessed GRE, and, in fact, at relatively higher concentrations. Total GSL content in most crops were considerably reduced in shoots compared to that on seeds and sprouts except for in leaf mustard, which possessed relatively higher total GSL content than that in sprouts. However, the value observed in this study was within the range of fully matured floret/leaf parts described in previous reports [5,31,32]. 

### 2.4. Variation in GSL Concentration in Roots

In *Brassica* roots, the most abundant GSLs were ERU, BRA, and NAS, of which NAS was present in the highest quantity in almost all crops, ranging from 0.12 µmol·g^−1^ in radish to 57.76 µmol·g^−1^ in leaf mustard (Table 5). ERU was the second major GSL in broccoli, cabbage, radish, baemuchae, kale, and pakchoi and the concentration was higher than in shoots that suggests higher health beneficial value of root as ERU has several antioxidant effects [29]. In contrast, other GSLs showed different accumulation patterns depending on the crop type. Total GSL concentration ranged from 4.51 µmol·g^−1^ in radish to 73.61 µmol·g^−1^ in leaf mustard. Moreover, total GSL concentration was higher in roots than in shoots, which is mainly due to the relatively higher concentration of NAS in roots than in other tissues. This finding also indicates a higher nutritive value of *Brassica* roots as breakdown products of NAS are known to have potential beneficial effects on human health [33]. Likewise, total GSLs concentration in roots in all the crops in this study were relatively higher than in their fully matured edible parts, as described in previous reports [5,26,34,35].

**Table 2:** Glucosinolate concentration (µmol·g^−1^ dry weight) in seeds of nine *Brassica* vegetables.

Crops	IBE	PRO	EPI	GRA	SIN	GRE	NAP	BCN	ERU	BRA	NAS	Total GLS
Baemuchae	N/D ^x^	11.85 c	0.28 c	0.14 f	0.22 e	0.24 b	18.10 b	7.62 a	0.32 c	0.05 f	1.80 c	40.62 e
Broccoli	N/D	29.84 b	0.77 b	0.36 c	N/D	N/D	3.74 e	N/D	73.77 a	0.25 d	2.03 b	110.76 a
Cabbage	1.06 ^y^ b ^z^	53.53 a	1.46 a	3.30 a	11.39 d	N/D	5.40 d	N/D	28.91 b	0.07 e	0.47 g	105.58 b
Cauliflower	12.03 a	0.92 e	N/D	0.22 d	34.09 c	N/D	0.31 g	N/D	1.21 c	0.73 a	N/D	49.52 d
Chinese cabbage	N/D	13.19 c	0.26 cd	0.18 e	0.01 e	N/D	16.76 c	3.73 b	0.26 c	0.43 c	1.57 e	36.38 f
Kale	0.55 c	9.08 d	0.25 d	0.09 g	66.49 b	N/D	2.72 f	N/D	1.36 c	0.07 e	2.44 a	83.04 c
Leaf mustard	N/D	N/D	N/D	N/D	77.27 a	N/D	4.00 e	0.10 d	N/D	0.04 f	1.29 f	82.70 c
Pakchoi	N/D	0.64 e	0.02 e	0.04 h	0.07 e	N/D	102.43 a	2.66 c	0.17 c	0.01 g	1.71 d	107.75 ab
Radish	N/D	N/D	N/D	0.90 b	n/d	0.70 a	N/D	N/D	0.38 c	0.46 b	N/D	2.43 g

^x^ N/D: Not detected; ^y^ Values are the means of three independent replications; ^z^ Different letters within a column indicate statistically significant by Duncan’s multiple-range test at *p* ≤ 0.05.

**Table 3:** Glucosinolate concentration (µmol·g^−1^ dry weight) in sprouts of nine *Brassica* vegetables.

Crops	IBE	PRO	EPI	GRA	SIN	GRE	NAP	BCN	ERU	BRA	NAS	Total GSL
Baemuchae	N/D ^x^	8.65 d	0.15 d	N/D	N/D	0.12 a	7.09 b	4.76 a	0.47 d	0.08 e	2.75 b	24.06 f
Broccoli	N/D	28.45 b	0.82 b	2.04 b	N/D	N/D	2.00 d	0.11 e	123.67 a	1.05 b	4.05 a	162.19 a
Cabbage	2.07 ^y^ b ^z^	33.82 a	1.33 a	6.63 a	9.93 e	N/D	2.41 d	0.07 f	34.88 b	0.34 d	0.47 e	91.94 b
Cauliflower	12.77 a	0.50 g	N/D	0.31 c	33.9 b	N/D	0.21 fg	0.69 d	N/D	1.98 a	0.30 g	50.66 d
Chinese cabbage	N/D	10.59 c	0.19 c	N/D	N/D	N/D	3.93 c	1.61 b	N/D	0.37 d	0.72 d	17.42 h
Kale	0.94 c	4.74 e	0.11 e	0.17 c	55.56 a	N/D	0.95 e	N/D	1.15 c	0.79 c	1.57 c	65.96 c
Leaf mustard	N/D	2.74 f	0.10 e	0.04 c	12.01 d	N/D	26.24 a	0.80 c	0.13 e	0.13 e	0.38 f	42.56 e
Pakchoi	N/D	0.08 g	N/D	0.03 c	20.22 c	N/D	0.70 ef	N/D	N/D	0.04 ef	0.22 h	21.29 g
Radish	N/D	N/D	N/D	0.10 c	N/D	0.11 a	0.07 g	N/D	1.25 c	0.74 c	0.19 h	2.46 i

^x^ N/D: Not detected; ^y^ Values are the means of three independent replications; ^z^ Different letters within a column indicate statistically significant by Duncan’s multiple-range test at *p* ≤ 0.05.

**Table 4:** Glucosinolate concentration (µmol·g^−1^ dry weight) in shoot of nine *Brassica* vegetables.

Crops	IBE	PRO	EPI	GRA	SIN	GRE	NAP	BCN	ERU	BRA	NAS	Total GSL
Baemuchae	N/D ^x^	2.19 b	0.10 c	0.02 d	0.12 c	4.11 a	0.44 cd	1.62 b	0.07 cd	0.95 e	1.53 a	11.14 c
Broccoli	0.11 ^y^ d ^z^	0.17 fg	0.37 a	0.72 b	0.09 c	N/D	0.07 f	N/D	N/D	1.44 d	1.20 c	4.17 de
Cabbage	0.37 c	2.58 a	0.18 b	1.20 a	0.64 c	N/D	0.55 c	0.44 c	0.26 b	3.13 a	1.57 a	10.92 c
Cauliflower	1.21 b	0.18 f	0.12 c	0.08 d	1.22 c	N/D	N/D	0.12 e	N/D	2.56 b	0.81 d	6.28 d
Chinese cabbage	N/D	1.04 d	0.09 c	0.02 d	N/D	N/D	0.16e f	0.32 d	0.08 c	1.10 e	0.72 d	3.53 e
Kale	1.52 a	1.34 c	0.38 a	0.23 c	6.20 b	N/D	0.29 de	0.12 e	0.29 b	2.28 c	0.84 d	13.47 b
Leaf mustard	N/D	0.07 gh	0.11 c	N/D	52.00 a	N/D	7.65 b	0.41 c	0.28 b	0.70 f	0.54 e	61.76 a
Packchoy	N/D	0.51 e	0.09 c	0.07 d	0.09 c	N/D	8.24 a	3.10 a	0.60 a	0.42 g	1.38 b	14.48 b
Radish	N/D	0.01 h	N/D	0.02 d	N/D	0.43 b	0.06 f	N/D	0.13 c	0.24 g	0.31 f	1.20 f

^x^ N/D: Not detected; ^y^ Values are the means of three independent replications; ^z^ Different letters within a column indicate statistically significant by Duncan’s multiple-range test at *p* ≤ 0.05.

**Table 5:** Glucosinolate concentration (µmol·g^−1^ dry weight) in roots of nine *Brassica* vegetables.

Crops	IBE	PRO	EPI	GRA	SIN	GRE	NAP	BCN	ERU	BRA	NAS	Total GSL
Baemuchae	N/D ^x^	3.36 a	0.12 a	0.12 d	0.13 e	0.52 b	1.28 b	3.35 a	4.18 c	0.35 e	17.49 f	30.91f
Broccoli	N/D	0.29 e	0.09 b	0.40 c	0.10 e	N/D	0.18 f	N/D	10.9 8b	2.11 a	34.35 d	48.50 d
Cabbage	0.17 ^y^ c ^z^	1.47 b	0.13 a	0.71 a	1.19 d	N/D	0.91 c	N/D	10.65 b	1.03 c	51.78 b	68.04 b
Cauliflower	0.32 b	0.05 ef	0.09 b	0.01 e	2.58 c	N/D	0.14 f	N/D	0.29 f	0.75 d	52.08 b	56.30 c
Chinese cabbage	N/D	0.62 d	0.08 b	0.02 de	0.09 e	N/D	0.10 f	0.64 b	0.59 ef	1.28 b	33.25 d	36.68 e
Kale	0.35 a	0.78 d	0.10 b	0.50 b	6.83 b	N/D	0.78 d	N/D	16.65 a	0.87 cd	29.44 e	56.30 c
Leaf mustard	N/D	0.12 ef	0.09 b	N/D	12.94 a	N/D	2.05 a	0.21 d	0.06 f	0.39 e	57.76 a	73.61 a
Packchoy	N/D	1.05 c	0.09 b	0.04 de	N/D	N/D	0.48 e	0.39 c	2.84 d	0.43 e	45.44 c	50.76 d
Radish	N/D	N/D	N/D	0.08 de	N/D	2.93 a	N/D	N/D	1.14 e	0.23 e	0.12 g	4.51 g

^x^ N/D: Not detected; ^y^ Values are the means of three independent replications; ^z^ Different letters within a column indicate statistically significant by Duncan’s multiple-range test at *p* ≤ 0.05.

### 2.5. Overall Variation in GSL Concentration

Among the different tissues or growth stages, in most *Brassica* vegetables examined, the highest GSL concentrations were noted in seeds, which then gradually decreased in sprouts and other parts. However, individual GSL concentrations were not consistent across all tissues examined. These observations, in general, are in good agreement with previous reports of broccoli and Chinese cabbage [4,36,37], and this might be due to the result of dilution caused by tissue expansion as a consequence of the metabolism of GSLs [37,38,39]. Furthermore, almost all the crops exhibited higher concentrations of GSLs in sprouts than in shoots. Similar results were also observed in sulforaphane and sulforaphene (hydrolyzed products from GRA and GRE, respectively) contents in broccoli and baemuchae [39], respectively, which might be attributed to the dilution of individual GSLs across the stages of development. Likewise, similar to the previous report by Li *et al.* [40], relatively higher GSLs levels could also observed in sprouts than in leaves in radish. However, an opposite trend was noted in leaf mustard, which showed higher GSL concentration in its shoots than in sprouts; this might be due to the difference in the regulatory mechanism underlying the GSL biosynthetic pathway [41]. Total aliphatic GSL concentration decreased in shoots than in seeds and sprouts for all crops examined, except leaf mustard. In contrast, BRA (an indole GSL) in shoots was higher than that in seeds and sprouts that could be due to *de novo* synthesis of this GSL during plant growth [38].

Higher GSL concentrations were observed in roots than in shoots at the same developmental stages across all crops, which are in accordance with previous reports of radish and cabbage [35,42,43]. In addition, GSL distribution patterns and concentrations in roots and shoots differed, because both these tissues have different regulatory mechanisms of GSL biosynthesis and turnover [44]. The differences in GSL contents across shoots and roots were essentially due to the difference in the contents of some specific GSLs such as ERU and NAS. In accordance with Bellostas *et al.* [4] and Velasco *et al.* [45], all the crops examined possessed relatively higher concentrations of aliphatic GSLs in their seeds, sprouts, and shoots, and their composition varied depending on the crop type and the tissues. Allelic variation in the genes encoding key regulatory enzymes at key points in the GSL pathway could also result in these differences [45]. Aliphatic GSLs constituted more than 85% of total GSLs in the seeds and sprouts of most crops, whereas they constituted 37%–98% of total GSLs in shoots.

However, among the aromatic GSLs, NAS was more dominant in the root than in other plant parts, except in radish. Similarly, higher NAS concentrations were also previously recorded [22,35,43] in cabbage and pakchoi roots, and the root GSLs exhibited higher variation than shoot GSLs. The higher total GSL concentrations and greater variation in their content in roots might be attributed to the higher survival pressure of roots because the roots endure numerous pathogens that are abundant in the soil environment. The different GSLs found in higher amount in the root may act as defensive compounds to deter invasion by these soil organisms [44]. PRO, one of the major GSLs in seeds and sprouts in most of the crops (except in pakchoi, leaf mustard, and radish) was present in relatively low concentrations in other parts examined. NAP, which was present in most of the crops and their respective tissues/developmental stages, was also present at the highest concentrations in seeds. However, radish and leaf mustard showed some unusual GSL content distribution. Furthermore, ERU was exceptionally lower or not found in shoots compared to other tissues in almost all crops examined. BRA showed opposite trend of accumulation with the highest content in shoot, which was gradually decreased in roots, nine-day-old sprouts, and was the lowest in seeds in most crops. Similarly, higher BRA content was also previously reported in mature tissues of *Arabidopsis thaliana* [46], likely due to the *de novo* synthesis of BRA with growth of these crops [38,47]. The NAS content was high in roots than in seeds, sprouts, and shoots in most crops examined, which resulted in higher levels of total GSL in roots. Such a remarkable variation in GSLs content and profiles within different tissues of a crop and among the different crops reflect variations in the control mechanisms underlying GSL biosynthetic pathways in different tissues of a single plant or might result from alternation in substrate availability [41] or selective catabolism of certain GSLs, which has previously been observed in other species belonging to the order *Capparales* [38,46,47]. To our knowledge, this is the first report of tissue distribution of several *Brassica* crops with accurate and speedy quantification (with in 18 min) using authentic 12 GSLs standards in UPLC while most of the other previous reports were based on the response factor or LC-MS based method.

### 2.6. Correlationships among GSLs

To understand the distribution patterns of the various GSLs and the composition content of total GSLs identified in *Brassica* crops, we evaluated the correlationships among different GSLs regardless of plant genotypes and the tissue examined (Table 6). All GSLs exhibited either significantly positive or no correlationship in that only aliphatic GSLs showed significant positive correlations, which is due to the biosynthetic relationships among aliphatic GSLs [48]. PRO, EPI, GRA, and ERU showed significantly positive correlationships with each other as well as with total GSLs. PRO, which was present in most of the crops and their parts in considerable amounts, exhibited the highest positive correlationship (*p* ≤ 0.001) with EPI (*r* = 0.944 ***), which was followed by GRA (*r* = 0.709 ***) and ERU (*r* = 0.649 ***). Furthermore, these GSLs also showed strongly positive correlationship with each other because all of these GSLs are 4 carbon aliphatic GSLs and follow quite similar biosynthetic pathway [15]. NAP and BCN also showed positive correlationship (*r* = 0.356 *, *p* ≤ 0.05) with each other. ERU exhibited highest positive correlation with total GSLs (*r* = 0.701 ***), which was followed by PRO, EPI and GRA. In contrast, other aliphatic GSLs such as IBE, SIN, and GRE showed no relationships with total GSLs. Similarly, none of the aliphatic GSLs showed significant correlations with indole GSL (BRA) and aromatic GSL (NAS) as they are derived from different amino acid precursors [15]. Furthermore, BRA and NAS showed no correlation with total GSLs, which might be because these GSLs were present in very minute quantities. However, a strong positive correlationship could be observed between NAS and total GSLs content in root parts (data not shown) that was due to the exceptionally higher content of NAS in roots.

## 3. Experimental Section

### 3.1. Plant Materials, Cultivation, and Sampling

Nine commercial *Brassica* vegetables were used in this study: baemuchae (cv. BB no.1), broccoli (cv. greendom), cabbage (cv. rudia), cauliflower (cv. snowdream), Chinese cabbage (cv. Seoul), kale (cv. matjjankale), leaf mustard (cv. akiobatacana), pakchoi (cv. chingen sai), and radish (cv. chunglyongmu). Seeds of cauliflower, broccoli, cabbage, and leaf mustard were obtained from Takii Co. (Kyoto, Japan); seeds of baemuchae, kale, and pakchoi were obtained from Asia Seed Co. (Seoul, Korea); and seeds of radish and Chinese cabbage were obtained from Koregon Co. (Seoul, Korea) and Syngenta Co. (Auckland, New Zealand), respectively. All vegetables were grown in a greenhouse at Chonbuk National University, Jeonju, Korea (50 m altitude, 35°49ʹN and 127°09ʹE). Seeds were sown on 5 September 2013 and tissue samples were harvested at two stages. The first harvests was performed 9 days after sowing, while the second harvest was performed either at 55 or 81 days after sowing, depending upon the optimal growth for harvest of the edible plant parts. Baemuchae, kale, leaf mustard, and radish were harvested at 55 days after sowing, whereas the other vegetables were harvested at 81 days after sowing. After harvest, whole plant parts from the first harvests were used for the study, and second-harvest samples were divided into shoots and roots. All the tissue samples were freeze-dried, ground into fine powder, and stored at −20 °C until GSL analysis.

**Table 6:** Overall correlation coefficients among glucosinolates identified in nine *Brassica* vegetables.

Glucosinolates	PRO	EPI	GRA	SIN	GRE	NAP	BCN	ERU	BRA	NAS	Total GSLs
IBE	−0.043	−0.057	0.063	0.290	−0.102	−0.105	−0.125	−0.069	0.234	−0.159	0.052
PRO		0.944 ***	0.709 ***	−0.070	−0.123	−0.016	0.030	0.649 ***	−0.257	−0.221	0.595 ***
EPI			0.831 ***	−0.101	−0.147	−0.067	−0.082	0.626 ***	−0.103	−0.172	0.546 ***
GRA				−0.075	−0.104	−0.082	−0.180	0.442 **	−0.019	−0.126	0.397 *
SIN					−0.170	−0.084	−0.246	−0.145	−0.165	−0.188	0.301
GRE						−0.095	0.050	−0.105	−0.080	−0.143	−0.295
NAP							0.356^*^	−0.074	−0.282	−0.148	0.289
BCN								−0.174	−0.286	−0.158	−0.120
ERU									−0.039	−0.051	0.701 ***
BRA										0.063	−0.270
NAS											0.163

*, **, *** Correlationships is significant at *p* ≤ 0.05, 0.01 and 0.001 respectively.

### 3.2. GSL Analyses

Sample preparation and GSL analyses were performed according to a modified version of the method described by Lee *et al.* [49]. Briefly, freeze-dried powder samples (0.1 g) were extracted with 2 mL of boiling methanol (70%) for 20 min and centrifuged at 12,000 rpm for 10 min at 4 °C. Thereafter, the pellet was re-extracted once more and the supernatants were pooled. The crude GSL extract was loaded onto a Mini Bio-Spin chromatography column (Bio-Rad Laboratories, Hercules, CA, USA) containing 0.5 mL of DEAE-Sephadex A 25 anion exchange resin, which was pre-activated with 0.1 M sodium acetate (pH 4.0). Next, desulfation was carried out by the addition of 200 µL of purified aryl sulfatase (EC 3.1.6.1, type H-1 from *Helix pomatia*). The column was capped and allowed to stand at room temperature for 24 h, and the desulfo-GSLs were eluted with 1.5 mL distilled water, filtered through a 0.2-µm syringe filter, injected into H-Class UPLC system (H-Class, Waters Co., Milford, MA, USA) using an Acquity UPLC^®^ BEH- C18 column (1.7 µm, 2.1 × 100 mm; Waters Co.), and its absorbance spectrum was measured at 229 nm with a photodiode array (PDA) detector. Solvent A (100% distilled water) and solvent B (20% acetonitrile in water) were used for the elution of compounds at the flow rate of 0.2 mL·min^−1^. The gradient programs were as follows: a linear step from 1% to 99% of solvent B within 6 min, followed by constant conditions for up to 10 min, and then a quick dropdown to 1% of solvent B at 12 min, and isocratic conditions of 1% of solvent B up to 18 min.

Authentic standards of GSLs were desulfated and used for the identification and quantification of the peaks. Concentrations of individual desulfo-GSLs were determined from the experimental peak area by analytical interpolation in a standard calibration curve of each desulfo-GSL across different ranges depending upon the GSLs and were expressed as micromoles per gram (µmol·g^−1^) of dry weight.

### 3.3. Authentic Standards and Chemicals

Twelve GSL standards, including glucoiberin (IBR), progoitrin (PRO), epiprogoitrin (EPI), glucoraphanin (GRA), glucoraphenin (GRE), sinigrin (SIN), gluconapin (NAP), glucobrassicanapin (BCN), glucoerucin (ERU), glucobrassicin (BRA), glucobarbarin (BAR), and gluconasturtiin (NAS), were purchased from Cfm Oskar Co. (Marktredwitz, Germany). Diethyl aminoethyl (DEAE), Sephadex-A25, and aryl sulfatase (EC 3.1.6.1, type H-1) from *H. pomatia* were purchased from Sigma-Aldrich (St. Louis, MO, USA). Other chemicals, including acetonitrile (HPLC grade), and formic acid (ACS reagent), were purchased from J.T. Baker (Phillipsburg, NJ, USA).

### 3.4. Statistical Analysis

Independent measurements in triplicate were used for each sample in all statistical analyses. To determine concentration differences among vegetables and their respective tissues, a one way analysis of variance (ANOVA), followed by Duncan’s multiple-range test (DMRT), were performed at a significance level of α = 0.05, using SAS^®^ 9.2 software (SAS Institute Inc., Cary, NC, USA, 2013).

## 4. Conclusions

The present study describes the accumulation patterns of individual GSLs in different tissues of nine *Brassica* crops. Our results indicate that the content and composition of GSLs in *Brassica* vegetables depend on the tissue type as well as the plant’s genotype. The total GSL concentration was the highest in seeds followed by that in sprouts, roots, and shoots. Collectively, our results highlight the distribution of different GSLs within the different tissues of a plant and among the different *Brassica* crops. The relatively high contents of aliphatic GSLs (IBE, PRO, EPI, GRA, SIN, GRE, NAP, BCN, and ERU) in seeds and sprouts, and that of aromatic GSL (NAS) in root parts suggest the higher nutritive value of these tissues. In addition, the relatively high content of NAS in the roots of all the crops examined suggests that the roots of these *Brassica* vegetables could be used for the extraction of GSLs in nutraceutical industry.
